# Inflammatory CD11b^+^ Macrophages Produce BAFF in Spleen of Mice Infected with *Leishmania donovani*

**DOI:** 10.3390/pathogens13030232

**Published:** 2024-03-06

**Authors:** Kazuki Nagai, Wataru Fujii, Junya Yamagishi, Chizu Sanjoba, Yasuyuki Goto

**Affiliations:** 1 Laboratory of Molecular Immunology, Graduate School of Agricultural and Life Sciences, The University of Tokyo, Tokyo 113-8657, Japan; kazuki-nagai007@g.ecc.u-tokyo.ac.jp (K.N.); asanjoba@g.ecc.u-tokyo.ac.jp (C.S.); 2 Laboratory of Biomedical Science, Graduate School of Agricultural and Life Sciences, The University of Tokyo, Tokyo 113-8657, Japan; awtrfj@g.ecc.u-tokyo.ac.jp; 3 Research Center for Food Safety, Graduate School of Agricultural and Life Sciences, The University of Tokyo, Tokyo 113-8657, Japan; 4 International Collaboration Unit, International Institute for Zoonosis Control, Hokkaido University, Sapporo 001-0020, Japan; junya@czc.hokudai.ac.jp

**Keywords:** visceral leishmaniasis, *Leishmania donovani*, B-cell-activating factor (BAFF), spleen, CD11b, macrophage

## Abstract

Visceral leishmaniasis (VL) is an infectious disease caused by parasitic protozoa of the genus *Leishmania* and manifests clinical symptoms such as splenomegaly, hepatomegaly, anemia, and fever. It has previously been shown that B-cell-activating factor (BAFF) is involved in splenomegaly during VL. Although BAFF is known to be expressed by a variety of cells, the mechanism of elevated BAFF expression in VL is not clear. In this study, we aimed to identify BAFF-producing cells in the spleens of mice infected with *Leishmania donovani*. Splenocytes of *L*. *donovani*-infected mice showed elevated BAFF expression compared to that of naive mice. In the infected spleen, the number of both CD11b^+^ and F4/80^+^ cells increased, and the major BAFF-producing cells were CD11b^+^ cells, which did not serve as host cells of *Leishmania*. Immunohistochemical/immunofluorescent staining of spleens of infected mice revealed that the increased CD11b^+^ cells were primarily MRP14^+^ mononuclear cells. Together, these results suggest the increased BAFF expression in the spleen of *L*. *donovani*-infected mice involves a recruitment of inflammatory macrophages distinct from host macrophages for the parasites.

## 1. Introduction

Visceral leishmaniasis (VL) is caused by *Leishmania donovani* and *L*. *infantum*, which proliferate within macrophages in the spleen, liver, and bone marrow of mammalian hosts. VL is primarily distributed in East Africa, South Asia, South America, and the Mediterranean region, with an estimated 50,000 to 90,000 of new cases each year (WHO, 2023). VL is characterized by clinical manifestations such as fever, weight loss, hepatosplenomegaly, anemia, and hypergammaglobulinemia. Pathogenesis of these symptoms has not been fully understood, and the elucidation may lead to the development of novel treatment and prevention options for VL.

B-cell-activating factor (BAFF), also known as BLyS, TALL-1, and TNFSF13B, is a cytokine that regulates survival and the activation of B cells [1,2]. BAFF induces the expression of Bcl-2, which inhibits the apoptosis of B cells, and the survival and proliferation of B cells is promoted [3,4,5]. The elevation of BAFF is observed in patients with several diseases that involve B cells such as autoimmune diseases [6], and the over-expression in BAFF-transgenic mice is known to result in a larger spleen than that of WT mice [7,8], like splenomegaly in experimental VL model mice [9]. The study using BAFF KO mice demonstrated that BAFF is involved in B cell hyper-activation and exacerbates splenomegaly during *L*. *donovani* infection in mice [10]. In fact, an increase in serum BAFF levels in VL patients can be found [11]. It is essential to reveal the molecular mechanisms of how BAFF is upregulated and contributes to those symptoms during VL in order to manage the disease.

Anti-BAFF antibodies are used in the treatment of systemic lupus erythematosus (SLE), and treatment targeting BAFF has been reported to be effective in relieving symptoms [12,13]. However, anti-BAFF antibodies including belimumab may serve as immunosuppressive agents, and their use may cause more serious infections and other bacterial and viral infections. In leishmaniasis, BAFF signaling has been shown to activate macrophages to protect against *L*. *major* infection in vitro [14]. On the other hand, in vivo experiments demonstrated that the number of parasites in BAFF-KO mice is unchanged in the spleen but increased in the liver compared to WT mice [10]. Also, total mRNA levels for *Tnfsf13b* coding BAFF are not significantly different between uninfected and infected spleen (unpublished). Therefore, systemic suppression of BAFF is not necessarily suitable for the treatment of VL. It is important to understand the local mechanisms of BAFF elevation, especially BAFF-producing cells, during infection.

Increased BAFF production occurs in some diseases, but various cells and tissues produce BAFF. SLE is characterized by activation of autoantibody-secreting B cells [15], and T cells from SLE patients produced a higher amount of BAFF than T cells from normal controls under basal conditions [16,17]. On the other hand, BAFF-producing neutrophils, monocytes, conventional dendritic cells (DCs), and T cells were all expanded in the spleens in spontaneous mouse lupus models using *Tlr7* transgenic mice and *Sle1* transgenic mice [18]. Organ-specific autoimmune diseases are associated with locally elevated BAFF. In rheumatoid arthritis patients, expression of BAFF from monocytes, T cells, and B cells in synovial tissues is increased [19]. In patients with Sjögren’s syndrome, the expression of BAFF from macrophages and T cells in salivary glands is increased [20].

In infectious diseases, BAFF-producing cells depend on the site of infection and inflammation. For example, astrocytes produce BAFF within the brain following encephalitis induced by cytomegalovirus [21] and neurotropic coronavirus [22]. Airway epithelial cells infected by respiratory syncytial virus increase BAFF expression [23]. Infection with a virulent strain of *Salmonella typhimurium* increased BAFF expression in splenic conventional DCs and inflammatory Ly6C^hi^ monocytes/DCs 4 days post-infection [24]. And this *Salmonella* infection induces down-regulation of BAFF expression levels, but the number of BAFF-producing neutrophils increases in the spleen [24].

Since BAFF-producing cells are disease-specific, it is necessary to identify which cells are BAFF-producing cells in VL. It is also necessary to consider not only the expression levels but the numbers of BAFF-producing cells to reveal mechanisms of BAFF up-regulation. In this study, we characterized BAFF-producing cell in the spleen during *L*. *donovani* infection.

## 2. Materials and Methods

### 2.1. Mice and Parasites

Female BALB/cA mice were purchased from Japan Clea, Tokyo, Japan. The mice were used for experiments at the age of 6–8 weeks. This animal experiment was reviewed and approved by an institutional animal research committee at the Graduate School of Agricultural and Life Sciences, The University of Tokyo (No. P22-057). Promastigotes of *L*. *donovani* (MHOM/NP/03/D10; gifted from National BioResource Project at Nagasaki University [25]) were cultured in Medium 199 (Thermo Fisher Scientific, Waltham, MA, USA) supplemented with 10% heat-inactivated fetal bovine serum (HI-FBS; Thermo Fisher Scientific) at 25 °C.

### 2.2. Experimental Infection

Mice were infected with 1 × 10^7^ promastigotes of *L*. *donovani* by intravenous injection into the tail vein and sacrificed at 6 months after infection. Spleen and liver were harvested from 5 naïve mice and 5–6 infected mice. Blood was collected by cardiac puncture and serum was collected by centrifugation of the blood at 5000× *g* for 15 min at 4 °C. Splenocytes of naive and infected mice were isolated through cell strainer (Corning, Corning, NY) after incubation of spleen with 1mg/mL collagenase, type IV, from *Clostridium histolyticum* (Sigma-Aldrich, St. Louis, MO, USA), and red blood cells were lysed with lysing solution (Sigma-Aldrich). The cells were washed with 1% HI-FBS/PBS and the number of cells counted by Countess 3 Automated Cell Counter (Thermo Fisher Scientific).

### 2.3. Flow Cytometric Analysis

Splenocytes were incubated with PE-conjugated anti-CD11b monoclonal antibody or anti-F4/80 monoclonal antibody (BD Pharmingen, Franklin Lakes, NJ, USA). At least 50,000 cells per sample were analyzed on the BD FACSVerse^TM^, and data analysis was performed using BD FACSuite^TM^ software Version 1.0.3. The splenocytes were gated in the FSC-SSC dot plots and the percentage of CD11b+ or F4/80+ cells were calculated.

### 2.4. Magnetic Separation of Splenocytes

Splenocytes were separated by 3 cell surface markers: CD3ε, CD11b, and F4/80. For CD3ε or CD11b separation, 10 μL of biotinylated hamster anti-mouse CD3ε or biotinylated rat anti-mouse CD11b (BD Biosciences, San Diego, CA, USA) was added to 2 × 10^7^ splenocytes suspension in 1% HI-FBS/PBS. The cell suspension after 15 min of incubation on ice was centrifuged at 500× *g* for 5 min and washed with 1% HI-FBS/PBS. Then, 20 μL anti-biotin microbeads (Miltenyi Biotec, Bergisch Gladbach, Germany) were added to cell suspension and it was incubated for 15 min on ice. For F4/80 separation, 20 μL anti-F4/80 microbeads (Miltenyi Biotec) were added to 2 × 10^7^ splenocytes suspension, and it was incubated for 15 min on ice. Separation was performed using MiniMACS^TM^ separator (Miltenyi Biotec) and MS column (Miltenyi Biotec). Before cell separation, MS column was placed on the separator, and rinsed with 500 μL of the buffer. The labeled cells were applied onto the rinsed MS column, and washed with the buffer, and then flow-through was collected as CD3ε^−^, CD11b^−^, and F4/80^−^ cell population. After the column was removed from the separator, 1 mL of buffer was added, and then the fraction was collected with the buffer as CD3ε^+^, CD11b^+^, and F4/80^+^ cell population. The number of cells in each fraction was counted using Countess 3 Automated Cell Counter.

### 2.5. BAFF Measurement

BAFF concentrations in samples were measured by sandwich ELISA using Mouse BAFF/BLyS/TNFSF13B DuoSet ELISA (R&D Systems, Inc., Minneapolis, MN, USA), according to manufacturer’s instruction. Splenocytes were dissolved with 1 × 10^7^ cells/mL RIPA buffer, and the amount of BAFF per 10^7^ cells was calculated.

### 2.6. Immunohistochemical Analysis

To examine the expression of CD11b in spleen, immunohistochemical staining was performed. The paraffin-embedded tissues were sectioned at 4 µm thickness and dewaxed. The tissues were boiled in Tris-EDTA buffer (10 mM Tris Base, 1 mM EDTA solution, 0.05% Tween 20, pH 9.0). After blocking with Block Ace (DS Pharm., Osaka, Japan), rabbit anti-mouse CD11b (Abcam, Cambridge, UK) antibody was applied to the serial sections of spleens, and the sections were incubated for 1 h at room temperature and washed with PBS-T (0.05% Tween 20 in PBS). Then, biotinylated anti-rabbit IgG (Nichirei, Tokyo, Japan) was applied, and the sections were incubated for 1 h at room temperature and washed with PBS-T. Alkaline phosphatase-conjugated streptavidin (Nichirei) was applied, and the sections were incubated for 30 min at room temperature. After enzymatic color development was performed using 4-[(4-amino-m-tolyl)(4-imino-3-methylcyclohexa-2,5-dien-1-ylidene)methyl]-o-toluidine monohydrochloride (new fuchsine, Nichirei), the sections were counterstained with Mayer’s hematoxylin solution for 2 min and rinsed with tap water. In the immunohistochemically stained spleen sections, CD11b-positive cells were counted in around 10 random microscopic fields at 1000× magnification. At the same time, whether each cell was a mononuclear (MN) cell or polynuclear (PMN) cell was determined by nuclear morphology.

### 2.7. Immunohistochemical Analysis

An immunofluorescence assay was performed to characterize CD11b-positive cell as MRP8/14-positive cells. Briefly, the primary antibodies, rabbit anti-mouse CD11b (Abcam, Cambridge, UK), and goat anti-mouse MRP8 or MRP14 (Santa Cruz Biotechnology, Santa Cruz, CA, USA), were applied to tissues following the immunohistochemical staining procedure. Then, they were incubated with the secondary antibodies in the order Alexa fluor 546 conjugated donkey anti-goat IgG then Alexa fluor 488 conjugated goat anti-rabbit IgG (Thermo Fisher Scientific) for 1 h, and counterstained with Hoechst 33342 (Dojindo, Kumamoto, Japan). Fluorescence observation was performed with Keyence BZ-X800 (Keyence, Osaka, Japan).

### 2.8. Western Blotting

To characterize CD11b-positive fraction in magnetic separation of splenocytes, Western blot analysis of MRP14 and a leishmanial protein TSA [26] was performed. Sera from rabbits immunized six times with either recombinant TSA formulated in Freund’s incomplete adjuvant were used as anti-TSA antibody [27]. The cell lysate samples of 1 × 10^5^ cells per lane were separated by electrophoresis on an SDS-containing 15% polyacrylamide gel and transferred to polyvinylidene difluoride membrane (Roche, Mannheim, Germany). After blocking with 4% skimmed milk, samples were incubated with anti-MRP14 for 24 h or anti-TSA antibody for 1 h at room temperature, followed by probing with HRP-conjugated anti-goat IgG antibody or anti-rabbit IgG antibody, respectively. Bound antibodies were visualized using ECL (GE Healthcare, Buckinghamshire, UK). Chemiluminescence signals were detected using an iBright FL1500 Imaging System (Thermo Fisher Scientific) according to the manufacturer’s instructions.

## 3. Results

### 3.1. CD11b^+^ Cells in the Spleen-Produced BAFF during L. donovani Infection

**Figure 1:**
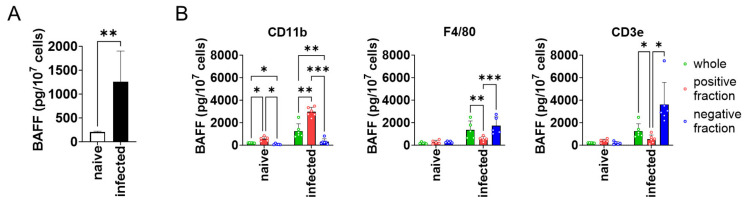
CD11b^+^ splenocytes highly expressed BAFF during *L*. *donovani* infection. (**A**) Mean + SD of BAFF protein amount per 10^7^ splenocytes of naïve or infected mice is shown. ** *p* < 0.01 by Kolmogorov–Smirnov test followed by unpaired *t* test. (**B**) Mean and SD of BAFF protein amount per 10^7^ cells are shown. Green bars show whole splenocytes. Red and blue bars show positive or negative fraction for CD11b/F4/80 CD3ε, respectively. Representatives of two independent experiments with similar results are shown. * *p* < 0.05, ** *p* < 0.01, *** *p* < 0.001 by two-way ANOVA followed by Tukey’s multiple comparisons test.

To see whether BAFF expression is higher in the spleen of *L*. *donovani*-infected mice compared with naïve mice, splenocytes were harvested from both naïve and infected mice and analyzed for BAFF expression. BAFF expression by the splenocytes of infected mice was six times higher than that of naïve mice (Figure 1A). Next, to characterize the BAFF-producing cells in the spleen of infected mice, MACS-based target cell separation was performed for CD3ε, CD11b, and F4/80, and the positive/negative fractions were examined for BAFF expression. CD11b^+^ cells showed higher expression of BAFF than CD11b^−^ cells (Figure 1B). On the other hand, F4/80^+^ and CD3ε^+^ cells showed lower expression of BAFF than F4/80^−^ and CD3ε^−^ cells, respectively (Figure 1B).

### 3.2. The Number of CD11b^+^ Cells in the Spleen Increased during L. donovani Infection

**Figure 2:**
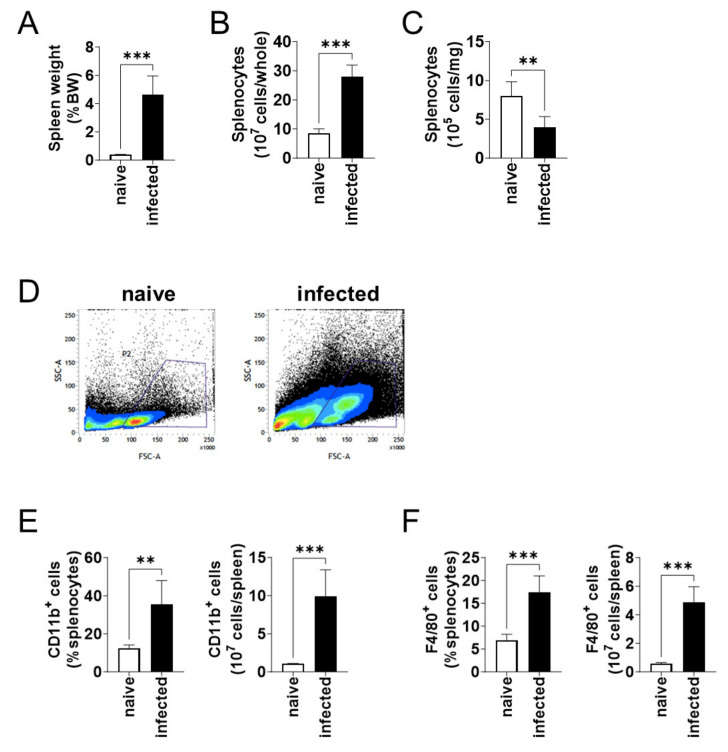
CD11b^+^ and F4/80^+^ cells increased in spleen during *L*. *donovani* infection. (**A**) Mean and SD of the spleen weights of each group are shown. (**B**) Mean + SD of the number of total splenocytes are shown. (**C**) Mean and SD of the number of splenocytes per 1 mg tissue are shown. (**D**) Representative profiles of the FSC-SSC dot plots of splenocytes from naïve and infected mice by flow cytometry. (**E**,**F**) Mean + SD of the percentage and the total number of CD11b^+^ (**E**) and F4/80^+^ (**F**) cells in the spleen are shown. Representatives of two independent experiments with similar results are shown. ** *p* < 0.01 and *** *p* < 0.001 by Kolmogorov–Smirnov test followed by unpaired *t* test.

At 6 months of infection, the body weight of naïve and *L*. *donovani*-infected mice were not different. On the other hand, the spleen weight per body weight of the infected mice was over 12 times larger than that of the naïve mice (Figure 2A). Although the enlarged spleen contained an increased number of splenocytes (Figure 2B), the number of splenocytes per weight decreased in the infected mice (Figure 2C). Flow cytometric analyses showed an increase in large cells (high FSC value) in the spleen of infected mice (Figure 2D). When the number of CD11b^+^ and F4/80^+^ cells was compared between the naïve and infected mice, an increase in both the proportion and the total number was found for both CD11b^+^ (Figure 2E) and F4/80^+^ (Figure 2F) cells in the infected spleen.

### 3.3. CD11b^+^ Mononuclear Cells Accumulated in the Spleen during L. donovani Infection

**Figure 3:**
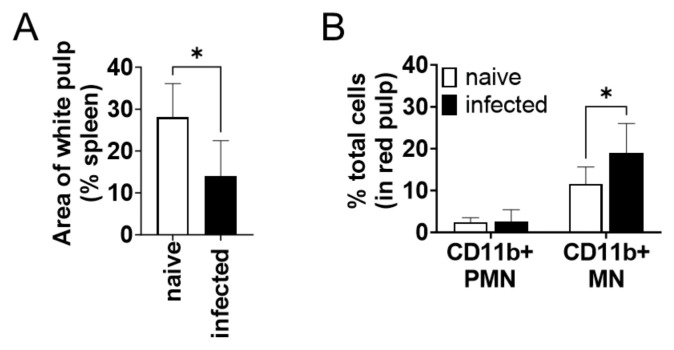
CD11b^+^ mononuclear cells in the spleen accumulated in the spleen during *L*. *donovani* infection. (**A**) Mean and SD of area proportion of white pulp in the spleen are shown. * *p* < 0.05 by unpaired *t* test. (**B**) Mean and SD of percentage of CD11b^+^ PMN and MN cells in red pulp are shown. Representatives of two independent experiments with similar results are shown. * *p* < 0.05 by two-way ANOVA followed by Tukey’s multiple comparisons test.

The CD11b^+^ cells increased in the spleen during infection were examined by microscopic observation of immunohistochemically stained tissues. The percentage of white pulp areas significantly decreased from 28 ± 8% to 14 ± 9% during infection (Figure 3A). Immunochemical staining revealed that almost all CD11b^+^ cells were present in the red pulp but not in the white pulp. The proportion of CD11b^+^ cells to the total cells in red pulps increased during infection (Figure 3B). To morphologically characterize CD11b^+^ cells increased in the spleen during infection, the number of CD11b^+^ cells was counted for each nuclear morphology: mononuclear (MN) and polymorphonuclear (PMN). The number of CD11b^+^ MN cells was significantly increased in the red pulp of the infected spleen but that of CD11b^+^ PMN cells did not change (Figure 3B). The CD11b^+^ MN cells in the red pulp had relatively wide cytoplasm, sharing morphological characteristics with macrophages and distinct from lymphocytes.

### 3.4. CD11b^+^ Cells in the Spleen during Infection Are MRP8/14-Positive and Are Distinct from Host Macrophages for L. donovani

**Figure 4:**
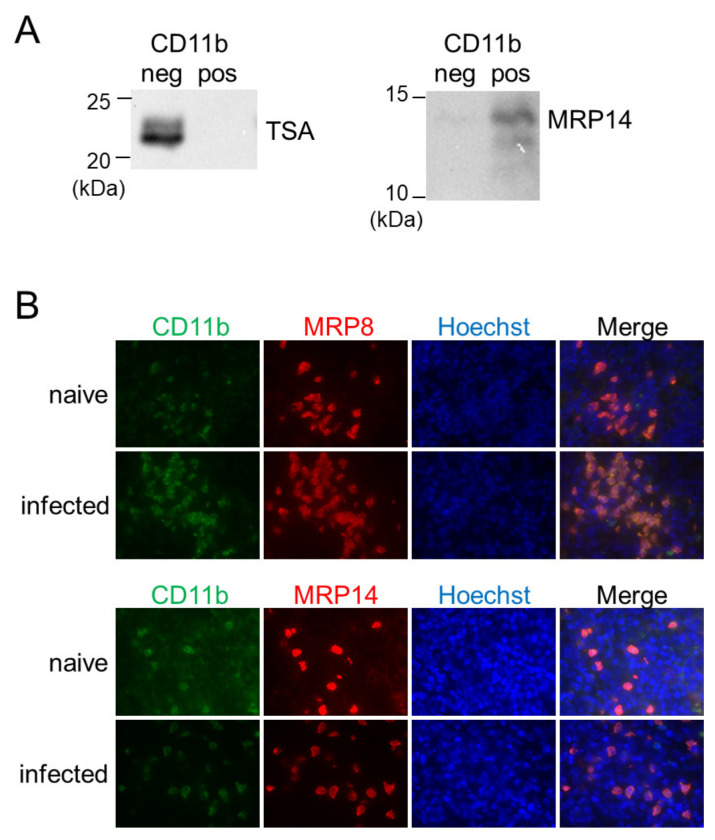
CD11b^+^ MN cells in the spleen during *L*. *donovani* infection were positive for MRP8 and MRP14 but not for the parasites. (**A**) The negative and positive fractions separated by MACS using anti-CD11b antibody were analyzed with Western blotting for expression of TSA or MRP14. Shown data are samples representative of five mice with similar results. (**B**) The spleens of naïve or infected mice were stained for CD11b, MRP8, MRP14, and Hoechst33324. Representatives of two independent experiments with similar results are shown.

In a previous study, we have reported that inflammatory macrophages expressing MRP8 and MRP14 accumulate in the spleen during *L*. *donovani* infection [28]. To reveal the relationship between the MRP8/14-expressing macrophages and the BAFF-producing CD11b^+^ MN cells, the CD11b-positive or -negative fraction of the splenocytes from *L*. *donovani*-infected mice was analyzed for expression of MRP14 by Western blotting. A band for MRP14 was detected in the CD11b-positive fraction but not in the CD11b-negative fraction (Figure 4A). Immunofluorescence staining of the *L*. *donovani*-infected spleen also demonstrated overlapped locations of cells expressing MRP8/14 and CD11b^+^ cells (Figure 4B). When the forementioned CD11b-positive or -negative fraction was investigated for the presence of *Leishmania* parasites by Western blotting using antileishmanial antibody, a band was detected in the CD11b-negative fraction but not in the CD11b-positive fraction (Figure 4A).

## 4. Discussion

The purpose of this study was to determine the mechanism by which BAFF, one of the factors causing splenomegaly in experimental VL [10], is elevated in the spleen during *L*. *donovani* infection. The results of this study suggest that the major population of BAFF-expressing cells in the infected spleen is CD11b^+^ inflammatory macrophages expressing MRP8 and MRP14, but not tissue-resident macrophages or activated T cells.

BAFF is involved in B cell homeostasis in a steady state [29,30] as well as the induction of humoral immunity during infections [31,32,33]. However, the source of BAFF may not be necessarily identical in the two distinct conditions. For example, Cremasco et al. demonstrated that fibroblastic reticular cells (FRCs) in the B cell zone constitute a chief source of BAFF in a steady state [34]. Another study utilizing reciprocal bone marrow chimeras with WT and BAFF-KO mice has revealed that BAFF from radiation-resistant stromal cells is completely sufficient to provide a necessary signal for B cell survival/maturation, whereas BAFF from bone marrow-derived cells is not at all sufficient to support normal B cell homeostasis [35]. Follicular dendritic cells (FDCs) are stroma cells located in the central region of primary follicles and in the light zone of germinal centers, and FDCs in lymph nodes express BAFF abundantly and they enhance B cell proliferation and retention in germinal center [2,36]. On the other hand, recent studies have demonstrated that BAFF from bone marrow-derived cells is involved in the pathogenesis of some infectious or autoimmune diseases. BAFF from neutrophils and conventional dendritic cells, not monocytes or macrophages, is responsible for T cell-independent antibody responses to infection with West Nile virus [37]. A similar case is found in *Salmonella typhimurium* infection, and the increase in B cells and plasma cells in the spleen during infection is also mediated by BAFF from neutrophils and conventional DCs [24]. In contrast, the increased number of mature B cells in a mouse model of SLE is dependent on BAFF from monocytes and conventional dendritic cells, but not neutrophils [18]. Our findings on CD11b^+^ inflammatory macrophages as the major source of BAFF in the spleen during VL adhere to findings in the latter cases of inflammatory conditions.

Here, we characterized the BAFF-expressing CD11b^+^ cells as inflammatory macrophages but not neutrophils, according to the expression of MRP8/MRP14 and the morphology of their nuclei (Figure 3B and Figure 4). In general, MRP8 and MRP14 are expressed by neutrophils, monocytes, and inflammatory macrophages [38,39,40]. It is of note that in some studies characterizing BAFF-expressing cells, MRP8 has been used as a neutrophil-specific marker for conditional knockout [18,24,37]. However, a study using the same strategy has revealed that MRP8-targeting conditional knockout affects not only neutrophils but also some parts of monocytes and splenic macrophages [41], in accordance with previous confirmation of MRP8 expression in those non-neutrophil cells [28]. Therefore, studies using the MRP8-targeting conditional knockout strategy may overlook the contribution of inflammatory monocytes and macrophages as a source of BAFF.

In contrast to a clear distinction of the BAFF-producing MRP8^+^ cells from neutrophils, we do not strictly distinguish inflammatory macrophages from inflammatory monocytes. Our terminology as inflammatory macrophages is more from a histological point of view, since we first identified an accumulation of MRP8^+^ mononuclear cells in the skin of *Leishmania major*-infected mice [42]. Later, we also found an accumulation of MRP8^+^ mononuclear cells in the spleen of *L*. *donovani*-infected mice [28]. Because of the shared expression of MRP8/MRP14 and shared morphological characteristics in the accumulated mononuclear cells between *L*. *major* and *L*. *donovani* infections, we defined MRP8^+^ mononuclear cells in the spleen of *L*. *donovani*-infected mice as inflammatory macrophages as well. Nevertheless, it has been reported that inflammatory monocytes are recruited to the spleen in the early stages of *Leishmania* infection [43,44]. Terrazas et al. have reported that these inflammatory monocytes have high expressions of MRP8 and MRP14, also known as S100A8 and S100A9, respectively [44]. Together, the BAFF-producing inflammatory macrophages expressing MRP8 and MRP14 observed in our study appear to be derived from these inflammatory monocytes. At the same time, there are some differences in the characters between the inflammatory monocytes in the early course of infection in previous studies and the inflammatory macrophages in the chronic course in the current study. For example, inflammatory monocytes in the early stages of infection can serve as the major host cells for *L*. *donovani* [44]. On the other hand, we found that macrophages expressing MRP8 and MRP14 are less infected compared with MRP8/MRP14-negative cells in the spleen chronically infected with *L*. *donovani* [28]. We also found that heavily infected macrophages have low expression levels of CD11b in the spleen of mice chronically infected with *L*. *donovani* [9]. These patterns were confirmed in the present study, as CD11b^−^ cells with no detectable MRP14 expression were more infected by *L*. *donovani* (Figure 3). In this study, BAFF-producing cells were identified as CD11b^+^ inflammatory macrophages. These cells are potential targets for therapy aimed at alleviating splenomegaly in VL. Inflammatory macrophages/monocytes are generally recruited from the bone marrow and blood [45]. If accumulation during infection can be inhibited, the supply of BAFF should be reduced and the symptoms of splenomegaly suppressed. Indeed, administration of antagonists against the chemokine receptor CCR2 suppresses the increase in spleen weight during *L*. *donovani* infection [44].

The coexistence of BAFF with MRP14 in those cells may accelerate splenomegaly in VL. MRP8 and MRP14 are known to be damage-associated molecular patterns that act as agonists for Toll-like receptor (TLR) 4 and activate TLR signaling [46,47,48]. The binding of MRP14 to TLR4 leads to the activation of NF-κB and the secretion of proinflammatory cytokines [49]. BAFF signals through the noncanonical NF-κB pathway, which contributes to B cell survival and differentiation and is also important for plasma cell maintenance and antibody production [50]. Activation of NF-κB by different pathways may synergistically enhance B cell survival and differentiation. A study using BAFF-transgenic mice has also shown that SLE-like disease required B cell-intrinsic TLR-associated MyD88 signaling [51]. Neutrophil-derived MRP14 causes the up-regulation of BAFF receptor on B cells and promotes the survival of multiple myeloma cells [52]. It is possible that MRP14 supports BAFF-mediated splenomegaly during *L*. *donovani* infection, since we have previously reported that splenomegaly during infection is mitigated in MRP14 KO mice [28]. To clarify the mechanism of splenomegaly, further studies are needed to understand the relationship between BAFF and endogenous inflammatory factors such as MRP8 and MRP14.

## Data Availability

Data presented in this study will be available from the corresponding author upon request.
